# Block Copolymer Membranes from Polystyrene-*b*-poly(solketal methacrylate) (PS-*b*-PSMA) and Amphiphilic Polystyrene-*b*-poly(glyceryl methacrylate) (PS-*b*-PGMA)

**DOI:** 10.3390/polym9060216

**Published:** 2017-06-10

**Authors:** Sarah Saleem, Sofia Rangou, Clarissa Abetz, Brigitte Lademann, Volkan Filiz, Volker Abetz

**Affiliations:** 1 Helmholtz-Zentrum Geesthacht, Institute of Polymer Research, Max-Planck-Str.1, 21502 Geesthacht, Germany; sarah.saleem@hzg.de (S.S.); sofia.rangou@hzg.de (S.R.); clarissa.abetz@hzg.de (C.A.) brigitte.lademann@hzg.de (B.L.); volkan.filiz@hzg.de (V.F.); 2 University of Hamburg, Institute of Physical Chemistry, Martin-Luther-King-Platz 6, 20146 Hamburg, Germany

**Keywords:** anionic polymerization, isoporous membrane, amphiphilic block copolymer, self-assembly, non-solvent induced phase separation

## Abstract

In this paper; we compare double hydrophobic polystyrene-*b*-poly(solketal methacrylate) (PS-*b*-PSMA) and amphiphilic polystyrene-*b*-poly(glyceryl methacrylate) (PS-*b*-PGMA) diblock copolymer membranes which are prepared by combining the block copolymer self-assembly in solution with a non-solvent induced phase separation (SNIPS). Diblock copolymers (i.e., PS-*b*-PSMA) were synthesized by sequential living anionic polymerization, whereas polystyrene-*b-*poly(glyceryl methacrylate) (PS-*b*-PGMA) were obtained by acid hydrolysis of the acetonide groups of the polysolketal methacrylate (PSMA) blocks into dihydroxyl groups (PGMA). Membrane structures and bulk morphologies were characterized by scanning electron microscopy (SEM) and transmission electron microscopy (TEM); respectively. The resulting PS-*b*-PGMA diblock copolymers produce an ordered hexagonal cylindrical pore structure during the SNIPS process, while membranes fabricated from the double hydrophobic (PS-*b*-PSMA) do not under similar experimental conditions. Membrane performance was evaluated by water flux and contact angle measurements.

## 1. Introduction

Block copolymers are well known for the formation of many microphase separated morphologies depending on the architecture of the block copolymer(s) involved, molecular weight, composition and thermodynamic properties [1,2,3]. Anionic living polymerization provides a way to the creation of well-defined block copolymers by the sequential addition of monomers. It is mostly used to synthesize block copolymers with controlled compositional and structural parameters such as molecular weight, narrow molecular weight distribution, copolymer composition, branching, and other architectural parameters. However, polymers from monomers with “active” protons (i.e., OH, SH, or NH groups) cannot be directly synthesized through this technique, as these react immediately with the initiator anions or the growing chain end [4]. To overcome this difficulty, either controlled radical polymerization can be employed, or protective groups are introduced into the monomeric unit blocking the reactive site during the course of anionic polymerization and these protected groups can be easily and readily cleaved afterwards to get the required functional groups [5]. Functional block copolymers have received extensive scientific and technological attention due to their potential applications in electronics, drug delivery [6], nano reactors [7], and smart materials [8,9]. Loeb and Sourirajan introduced for the first time the fabrication of synthetic membranes with advanced functionality via the non-solvent induced phase separation (NIPS) or so-called “phase inversion” process [10]. Later, the combination of the self-assembly of block copolymers and non-solvent induced phase separation (SNIPS) process allowed the preparation of isoporous membranes with high porosity and sharp molecular weight cut-off. In 2007, the first isoporous block copolymer membrane was reported to be cast from polystyrene-*block*-poly(4-vinylpyridine) (PS-*b*-P4VP) by utilizing the diblock copolymer self-assembly and non-solvent induced phase inversion process (SNIPS) [11]. Later on it was shown that different additives can support the membrane formation [12,13,14]. Additionally, post-modification of this type of membrane was done and led to superior properties, such as double stimuli-responsivity [15]. This concept was further extended to not only diblock copolymers, but also triblock terpolymers for the fabrication of integral asymmetric membrane via self-assembly SNIPS [16,17]. Blending of block copolymers was shown to be a facile way to tune the pore size of isoporous membranes [18]. A detailed study of the influence of the solvent on the structure formation in block copolymer membranes showed that the selectivity of the solvent plays a large role and also controls the suitable block copolymer concentration in the casting solution [19]. The development of isoporous membranes with improved functionality derived by postmodification of the block copolymer functional groups after membrane preparation can be promising for applications like water purification or protein separation [20]. Ultra- and nano-filtration membranes are mainly contaminated by biological substances which are prone to deposit on the surface as well as inside the pores of membrane [21]. It has been investigated that membranes with more hydrophilic surface hinder the attachment or accumulation of the foulants on the surface [22]. Nowadays, highly selective and permeable membranes with efficient antifouling properties are in high demand for ultrafiltration applications.

The most-studied hydrophilic segments in the field of amphiphilic block copolymers are poly(ethylene oxide) [17], poly(methacrylic acid) [23], and poly(2-hydroxy ethyl methacrylate) [24]. Poly(glyceryl methacrylate) (PGMA) is a potential alternative for the less hydrophilic 2-hydroxyethyl methacrylate (HEMA) due to the presence of one extra hydroxyl group (–OH) per repeating unit of the polymer in products such as contact lenses, drug delivery, and hydrogels. Poly(isopropylidene glycerol methacrylate)—commonly known as poly(solketal methacrylate)—acts as a precursor polymer of PGMA which was reported for the first time in 1990. Mori et al. reported the sequential anionic polymerization of styrene and solketal methacrylate (polystyrene-*b*-poly(solketal methacrylate), PS-*b*-PSMA) followed by the deprotection of acetonide groups to obtain PS-*b*-PGMA [25]. Zhang et al. used a combination of living anionic polymerization of allyl methacrylate (PAMA) and afterwards functionalization of the allyl side groups with osmium tetroxide to achieve PGMA [26]. Recently, the first attempt to prepare a membrane from this diblock copolymer was reported by Hahn et al., who used PS-*b*-PSMA for air brush spraying on a PVDF support membrane [27].

In this manuscript, for the first time double hydrophobic PS-*b*-PSMA and amphiphilic diblock copolymers of PS-*b*-PGMA are employed to cast integral asymmetric membranes via SNIPS. Previous work on spraying a solution of PS-*b*-PSMA followed by non-solvent-induced phase inversion did not result in a membrane with a uniform pore structure [27].

## 2. Materials and Methods

### 2.1. Materials

Tetrahydrofuran (THF) and *N*,*N*-dimethylformamide (DMF) were ordered from Th. Geyer. Styrene (Renningen, Germany), *sec*-butyllithium (*sec*-BuLi) and triisobutylaluminium (1 M in hexane) were purchased from Sigma-Aldrich (Darmstadt, Germany). *iso*-propylglycidyl methacrylate (SMA) was received from BASF SE (Ludwigshafen, Germany). THF was purified by distillation and titration with *sec*-butyllithium under argon atmosphere. Styrene (Sigma Aldrich, 99%) was purified from aluminium oxide and subsequently distilled from di-*n*-butylmagnesium (Sigma Aldrich, 1.0 M solution in heptane) under inert environment.

### 2.2. Instruments

The composition of diblock copolymers PS-*b*-PSMA and PS-*b*-PGMA were determined by proton nuclear magnetic resonance spectroscopy (^1^H NMR) on a Bruker Advance 300 NMR spectrometer (300 MHz, Bruker, Rheinstetten, Germany) with internal standard TMS (tetramethylsilane) using CDCl_3_ and DMF-*d7* as solvents. Polydispersity of the diblock copolymers and molecular weights of precursors were determined by gel permeation chromatography. The measurements were performed at 50 °C in dimethylacetamide (DMAc) and /or dimethylformamide (DMF) using 3 µm PSS SDV gel columns at a flow rate of 1.0 mL min^−1^ (VWR-Hitachi 2130 pump, Hitachi, Darmstadt, Germany). A Waters 2410 refractive-index detector (λ ¼ 930 nm) with a PS calibration was used.

Scanning electron microscopy (SEM) of the membranes was carried out on a LEO Gemini 1550 VP (Zeiss, Oberkochen, Germany) at a voltage of 3 or 5 kV. The samples were coated with 2.0 nm platinum. Cross-sections of the membranes were prepared while dipping the membranes in isopropanol, freezing in liquid nitrogen, and cracking. Average pore size values were determined using the software AnalySIS (Olympus Soft Imaging Solutions GmbH, Münster, Germany) on the basis of the SEM results. ImageJ 1.46 (Wayne Rasband, National Institute of Health, Madison, WI, USA) was used to determine the average porosity of our membranes.

Transmission electron microscopy (TEM) was carried out with an Tecnai G^2^ F20 (FEI, Eindhoven, The Netherlands) operated at 120 kV in bright-field mode. Thin sections (thin section thickness: 50 nm) were cut using a Leica Ultramicrotome EM UCT (Leica Microsystems, Wetzlar, Germany) equipped with a diamond knife (Diatome AG, Biel, Switzerland).

Dynamic contact angle measurements were measured on a KRUESS Drop Shape Analysis System DSA 100 (FEI part of Thermo Fisher Scientific, Kawasaki, Japan). Each sample was measured three times. The average error for both samples was ±1°.

### 2.3. Synthesis of (PS-b-PSMA) and Chemical Modification into (PS-b-PGMA)

All diblock copolymers were synthesized via sequential anionic polymerization under an inert atmosphere of high vacuum (10^−7^–10^−8^ mbar) and argon supply (Argon 7.0, Linde AG, Pullach, Germany). In the first step of polymerization, styrene was initiated with *sec*-BuLi (1.4 M solution in hexane) at −78 °C having THF as reactor solvent, and was left for 2 h to make sure that its polymerization was complete. An aliquot of polystyrene was sampled out from the reactor and terminated with degassed methanol for molecular characterization of the first block. Methacrylic ester containing monomer exhibits high electron affinity as compared to styrene, and it was added last in the polymerization procedure. Prior to the second monomer addition, 1,1-diphenylethylene (DPE) was added to end-cap the polystyrene macro-initiator and the temperature was maintained at −30 °C for half an hour. The flask was then cooled down to −78 °C before the addition of the purified SMA. The polymerization of the 2nd block was left to complete for 2 h. Degassed methanol was used to terminate the polymerization. All diblock copolymers were precipitated from their THF solution by a water/methanol mixture (80/20 *v*/*v*). To deprotect the acetonide group of PSMA, 1 N HCl was added drop-wise to a polymer in THF under continuous stirring. The reaction was left overnight to complete and then the polymer was precipitated in methanol. The hydrolyzed polymer was dried at 40 °C under vacuum and characterized by ^1^H NMR.

### 2.4. Membrane Formation

The dried block copolymer solutions were stirred for 24 h at room temperature and then cast directly with a homemade casting machine using a doctor blade with a gap height adjusted to 200 µm. For additional mechanical stability, block copolymer solutions were also cast on a polyester non-woven support.

## 3. Results and Discussion

### 3.1. Synthesis of Diblock Copolymers (PS-b-PSMA) (PS-b-PGMA)

**Figure 1:**
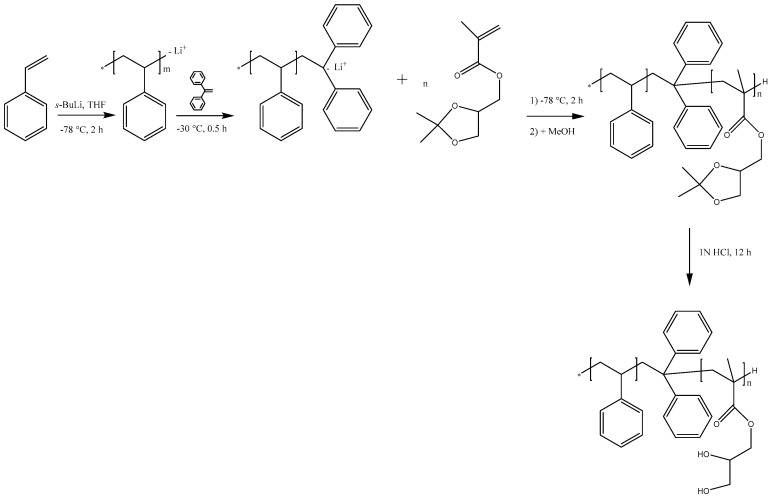
Route of synthesis for polystyrene-*b*-poly(solketal methacrylate) (PS-*b*-PSMA) and polystyrene-*b*-poly(glyceryl methacrylate) (PS-*b*-PGMA) by sequential anionic polymerization of styrene and isopropylidene glycerol methacrylate and subsequent hydrolysis. THF: tetrahydrofuran.

**Figure 2:**
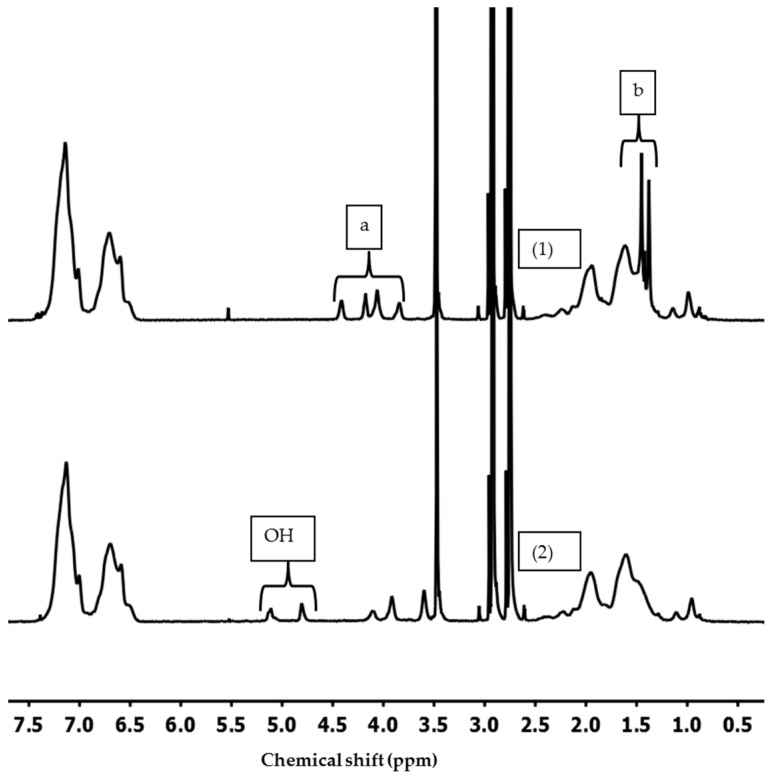
^1^H NMR spectra depicting the difference between the polymeric chains of PS_81_-PSMA_19_^170^ (1) and PS_85_-*b*-PGMA_15_^158^ (2) in DMF-*d7*.

PS-*b*-PSMA diblock copolymers were synthesized through living sequential anionic polymerization as depicted in Figure 1. The complete process of polymerization was optimized regarding monomer purification, monomer addition, block composition, and molecular weight. Figure 2 shows ^1^H NMR spectra of PS-*b*-PSMA (1) and the corresponding hydrolyzed PS-*b*-PGMA diblock copolymer (2). The 1,3-dioxolane ring in the PS-*b*-PSMA was readily cleaved by treating the block copolymer with 1 N HCl using THF as a solvent at room temperature overnight to generate the diol functions. The ^1^H NMR spectra (1) indicates that –OCH_2_CH (O)CH_2_(O) (3.84–4.41 ppm), –C(CH_3_)_2_(1.38–1.45 ppm) changed after modification of the PS-*b*-PSMA diblock copolymer. Two new peaks at (4.80, 5.11 ppm) appear in spectra (2) assigned to the two hydroxyl groups (–OH), and –C(CH_3_)_2_ peaks disappear completely which proves the complete hydrolysis of the PS-*b*-PSMA into the corresponding PS-*b*-PGMA. For a comparison between PS-*b*-PSMA and PS-*b*-PGMA diblock copolymers, DMF-*d7* was chosen as common solvent for ^1^H NMR; however, characterization of all the diblock copolymers was conducted in CDCl_3_ as given in Table 1 (supplementary information).

The subscripts show weight percentage of the individual blocks, whereas the superscript denotes the total number-averaged molecular weight *M*_n_ of the diblock copolymer calculated from ^1^H NMR and molecular weights of PS-precursors obtained from gel permeation chromatography (GPC, calibrated to PS). The amphiphilic nature of the block copolymers after hydrolysis did not permit GPC measurements using THF as an eluent, however; DMAc offers sufficient solubility for block copolymers before as well as after hydrolysis. Narrow monomodal molecular weight distributions were revealed by GPC using DMAc as eluent. In the case of PS_81_-PGMA_19_^128^, the poor solubility in DMAc led us to conduct GPC measurements in DMF. This illustrates the completion of the reaction without any side reactions. An increase in the number average molecular weight observed in PS-*b*-PGMA as compared to PS-*b*-PSMA diblock copolymers by GPC—even though the acetonide moiety was removed—confirmed similar results reported before by Frey et al. [28]. The hydrodynamic volume of the chemically modified diblock copolymer PS-*b*-PGMA increases in comparison with the starting diblock copolymer (PS-*b*-PSMA). This is due to the strong microphase separation tendency of the PS-*b*-PGMA. In order to check the behaviour of PS-*b*-PSMA and PS-*b*-PGMA in a common solvent, the hydroxyl groups of a PS-*b*-PGMA diblock copolymer were protected by the silylation reaction reported in literature by Hirao et al. [29] (see Figures S1–S5, Table S1 in Supplementary Materials).

In the process of asymmetric membrane formation, phase separation by spinodal decomposition and microphase separation by self-assembly of the diblock copolymer chains coexist. The microphase separation enhances the tendency of the viscous but still diluted phase to form pores of the hydrophilic block in a hydrophobic matrix, followed by the replacement of the remaining solvents from the casting solution with non-solvent, whereas the concentrated phase contributes in the structure formation of membrane. Due to direct contact with non-solvent bath, exchange of solvents occur fast enough on the top surface as compared to the bottom of the film.

The development of PS-*b*-PSMA and PS-*b*-PGMA integral asymmetric membranes with monodisperse surface pores via SNIPS demands uniform micelles in binary or ternary solvent systems prior to the precipitation step. Very few kinetic and thermodynamic studies of PSMA and PGMA have been published. In this study, the Hoy method [30] was used to measure solubility parameters of homopolymers of PSMA and PGMA, as displayed in Table 2.

**Figure 3:**
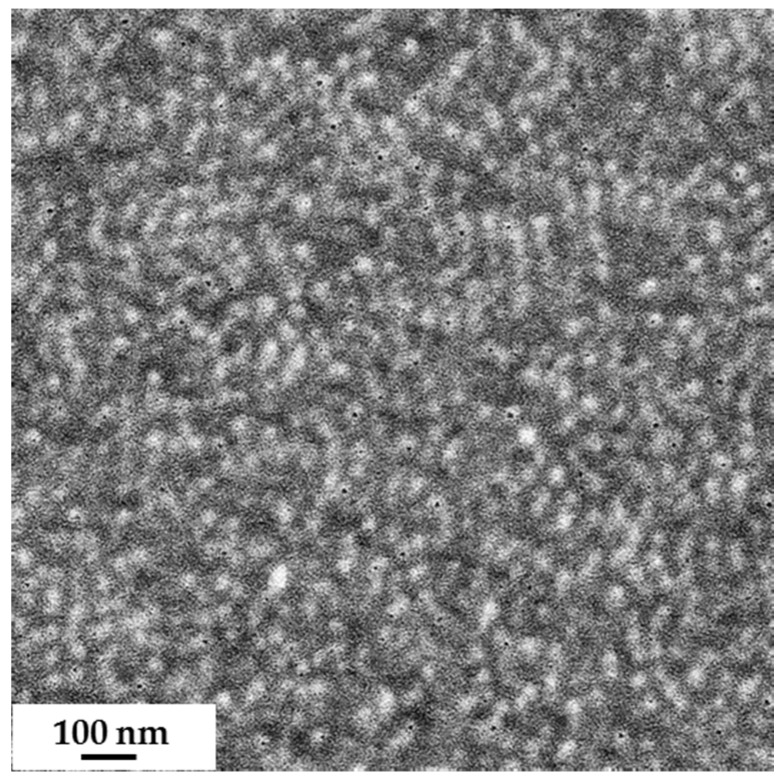
TEM image of an ultra-thin section of PS_81_-*b*-PSMA_19_^170^, film cast from 5 wt % polymer in THF; the diameter of the brighter spheres is approximately 25–30 nm.

According to the solubility parameters, the polymeric blocks of PS and PSMA should dissolve in THF, whereas PGMA should dissolve in more polar solvents like DMF. Due to the completely different nature of both PSMA and PGMA, we consider them individual systems and conducted a complete study on both polymers. The bulk morphology of the two block copolymers was studied by transmission electron microscopy following the commonly used preparation technique; i.e., the formation of thick films by slow drying of a polymer solution and annealing at a temperature above the glass transition temperatures of the blocks. Micelles of diblock copolymers were formed during film preparation. As PS-*b*-PGMA is only partially soluble in THF (which is a selective solvent for polystyrene, PS), the bulk morphology of this diblock copolymer was analyzed in DMF. For PS-*b*-PSMA diblock copolymer, THF was chosen as it is a suitable solvent for both blocks. Films were annealed very slowly from room temperature up to 120 °C, and ultrathin sections of approximately 50 nm were obtained at room temperature by ultramicrotomy. A TEM micrograph of the PS_81_-*b*-PSMA_19_^170^ diblock copolymer film is shown in Figure 3. Due to the sufficiently large electron density contrast between the two blocks no staining was necessary.

**Figure 4:**
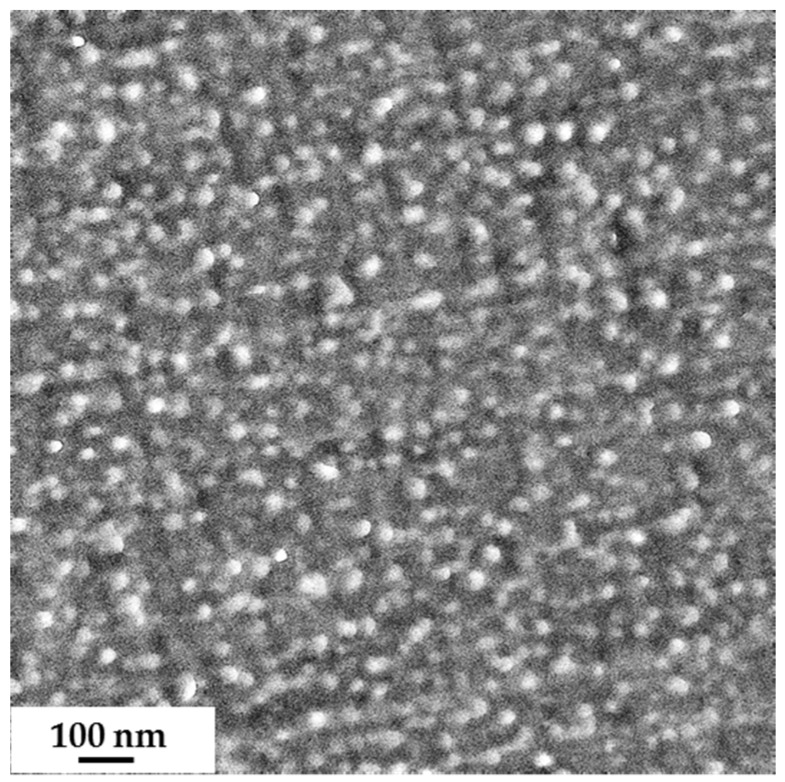
TEM image of an ultra-thin section of PS_81_-*b*-PGMA_19_^128^, film cast from 5 wt % diblock copolymer in DMF; the diameter of the brighter spheres is approximately 30–35 nm.

An ultrathin section of PS_81_-*b*-PGMA_19_^128^ film was also analyzed without staining due to the sufficiently large difference in the electron density of the two blocks (Figure 4). In the case of PS_81_-*b*-PGMA_19_^128^, a less-ordered morphology was observed with brighter spheres of PGMA in the darker polystyrene matrix. This may be due to the fact that DMF is a more selective solvent for PGMA, leading to a poorer solubility of the diblock copolymer.

### 3.2. Membrane Formation via SNIPS

The formation of the intended integral asymmetric membrane with hexagonally oriented porous cylinders on top of the spongy structure via SNIPS process is influenced by different parameters, such as evaporation time, solvent composition, and polymer concentration of the casting solution, which have to be further optimized. In the block copolymers of this study, the PS blocks from the matrix of the membrane, whereas the inner surface of the pores was formed by PSMA or PGMA in PS-*b*-PSMA and PS-*b*-PGMA, respectively.

**Figure 5:**
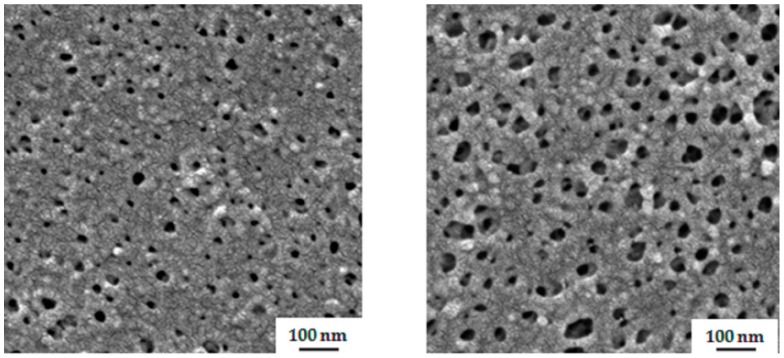
SEM images of the top surface of cast membranes from 24 wt % PS_76_-PSMA_24_^200^ from (**left**) THF/DMF 50/50 wt %, (**right**) THF/DMF 40/60 wt %. The time of evaporation before immersion into precipitant was 10 s.

Polystyrene-*b*-poly(solketal methacrylate) (PS-*b*-PSMA) is an unknown system in the context of SNIPS process, so different compositions of solvent mixtures were investigated. In Figure 5, the surface structure of membranes prepared from 24 wt % PS_76_-PSMA_24_^200^ polymer solution in THF/DMF with solvent compositions 50/50 wt % and 40/60 wt % shows in both cases a rather poor organization of pores. The evaporation time before immersion into non-solvent bath was 10 s.

**Figure 6:**
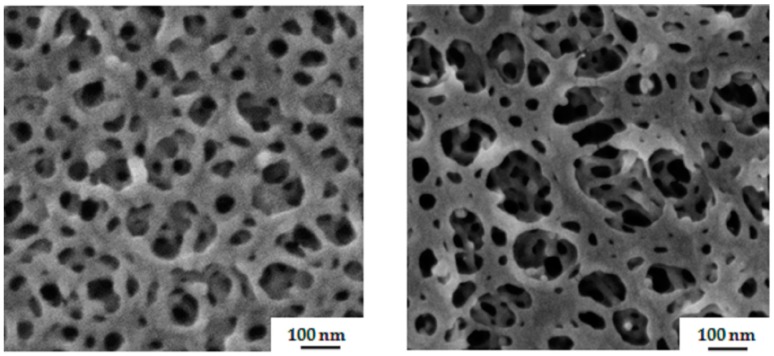
SEM topography images of the surface of the generated membrane from 25 wt % PS_76_-PSMA_24_^200^ in THF/DMF 30/70 wt % with evaporation time 10 s (**left**) and 20 s (**right**).

As shown in Figure 6 for an evaporation time of 10 s, a macroporous spongy-like structure formed by casting block copolymer solution in THF/DMF 30/70 wt %, whereas the surface structure of the membrane is getting more open with macropores which are not even interconnected at an evaporation time of 20 s.

**Figure 7:**
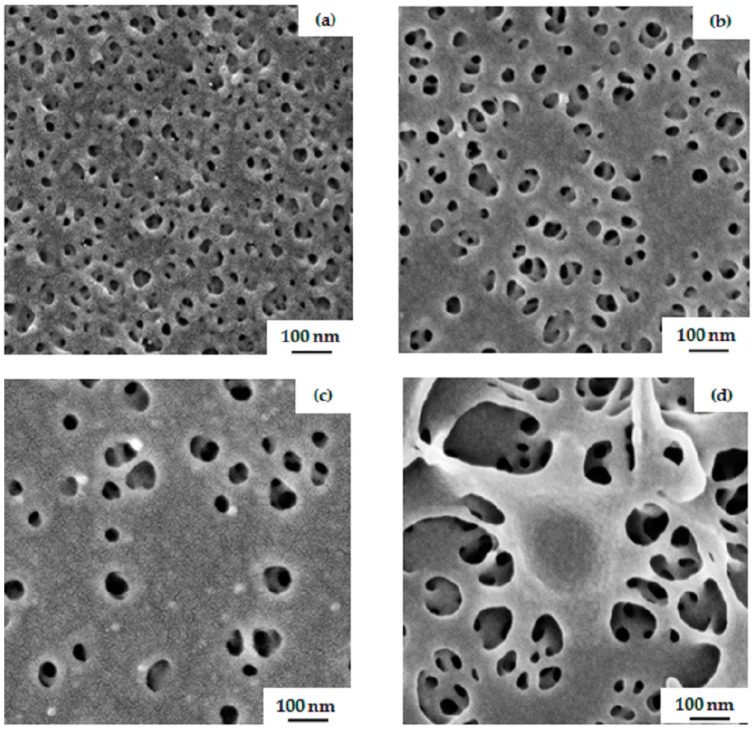
SEM images of surfaces of the membranes made from 24 wt% PS_81_-PSMA_19_^170^ in THF/DMF 50/50 wt %. Evaporation time before immersion: (**a**) 5 s, (**b**) 10 s, (**c**) 20 s, (**d**) 25 s.

During the SNIPS process, the evaporation time before immersion into a non-solvent bath affects the top surface structure of membrane. Figure 7 depicts the surfaces of the membranes prepared from a PS_81_-PSMA_19_^170^ 50/50 wt % solution THF/DMF for different evaporation times, namely 5, 10 and 20, 25 s. With an increase in evaporation time, the top surface with a very small number of macropores appeared together with a rather dense sub-structural morphology.

**Figure 8:**
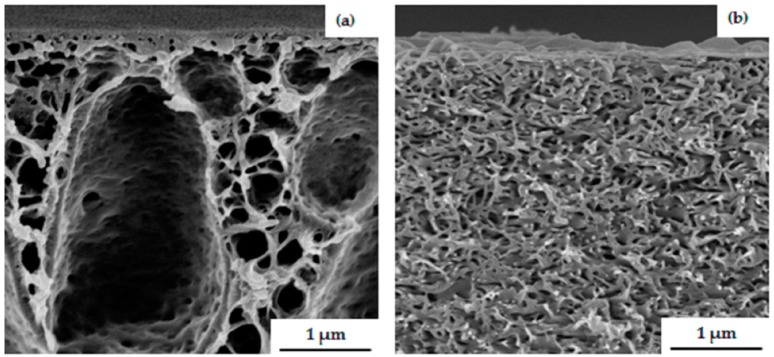
SEM images of the cross-section of the membrane prepared from 24 wt % PS_81_-PSMA_19_^170^ in THF/DMF 50/50 wt % at (**a**) 5 s, (**b**) 25 s time of evaporation.

The substructure of the PS_81_-PSMA_19_^170^ membrane shown in Figure 8a exhibits finger-like structures through almost the entire substructure of the membrane. Open finger-like structure with large voids appeared due to an instantaneous demixing of polymer-poor phase [31]; however, the substructure becomes more dense with increasing evaporation time, as shown in Figure 8b. While increasing the evaporation time the casting solution becomes viscous before its immersion into the non-solvent bath, due to evaporation of the more volatile THF. In addition, a longer evaporation time can lead to a decreasing concentration gradient of the diblock copolymer perpendicular to the surface, which also favors a parallel alignment of cylinders rather than the formation of standing cylindrical domains. An isoporous surface structure could not be obtained in any case, even by increasing the viscosity of the diblock copolymer solution. It can be concluded from surface and cross-sectional morphologies of these hydrophobic PS-*b*-PSMA diblock copolymers that this system still needs to be optimized with regard to self-assembly in combination with a non-solvent-induced phase separation process.

**Figure 9:**
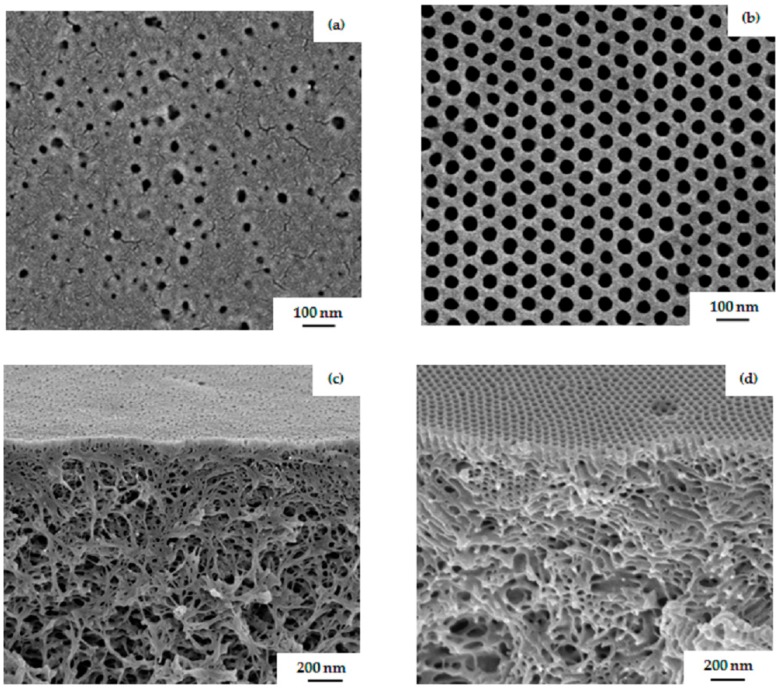
SEM topography images of membranes prepared from (**a**) 23 wt % PS_76_-PSMA_24_^135^ and (**b**) 23 wt % PS_81_-PGMA_19_^128^ in THF/DMF 50/50 wt %. The corresponding cross-section views for each case are shown in (**c**) and (**d**) images, respectively. The time of evaporation was 10 s.

Further, in this work, amphiphilic PS-*b*-PGMA diblock copolymers were used for casting integral asymmetric membranes via the SNIPS process. In Figure 9b,d, SEM images of the surface of a membrane prepared from 23 wt % PS_81_-PGMA_19_^128^ in THF/DMF 50/50 wt % under blade height of 200 µm show a regular pattern of hexagonally-oriented open pores with a sponge-like structure underneath. The time of evaporation before immersion into the non-solvent bath was 10 s. The pore diameter in this case was approximately 33 ± 2 nm. An ordered perpendicular cylindrical morphology was observed for an evaporation time window from 5–15 s; however, the isoporous structure of the block copolymer membrane collapsed after 20 s. When the same casting conditions are applied to the non-modified block copolymer PS_76_-PSMA_24_^135^, the surface structure shows very few open pores with a compact structure underneath, as shown in Figure 9a,c. As is to be expected, a remarkable difference between surface and substructure of the membranes indicates that the second block has a tremendous effect in the development of isoporous membrane. Before modification, it behaves completely differently, and is unable to form a regular hexagonal structure. In PS_76_-*b*-PSMA_24_^135^, both blocks are hydrophobic ,whereas after hydrolysis of the acetonide groups, an amphiphilic system is generated which has a tendency to strongly microphase segregate due to the hydrophilicity of the PGMA blocks, resulting in an open porous membrane structure. In case of the water-incompatible double hydrophobic system PS-*b*-PSMA, a closer dense membrane is obtained via phase inversion process similar to a hydrophobic homopolymer.

At this point it should be mentioned that the open porous membrane surface structure is a combined result of the strongly amphiphilic character of the block copolymer, which is a prerequisite to be very selective in the interaction with the involved solvents and the solvent-induced phase separation. Specifically for a solution system with two solvents, the less volatile but more polar solvent swells the polar minority block, while the more volatile but less polar solvent is selective for the less polar matrix-forming block, which is the one which vitrifies at the surface first due to the fast evaporation of this more volatile solvent. In the case we deal with a double hydrophobic block copolymer and using the aforementioned solvent system, we no longer have such a strong selectivity of the solvents, and therefore the formation of an isoporous surface structure is unlikely to occur. As the incompatibility of the different blocks is given by the product of segmental interaction parameter and the degree of polymerization, a larger degree of polymerization would be required to give the same degree of incompatibility for the unhydrolyzed diblock copolymer as compared to the hydrolyzed one. Therefore, if for a given hydrolyzed diblock copolymer a hexagonally-ordered porous membrane structure can be obtained, for the unhydrolyzed diblock copolymer a microphase separation is expected for higher degrees of polymerization. On the contrary, the viscosity would increase due to the higher molecular weight and this would also lead to other requirements for the evaporation time, again affecting the necessary solvent concentration gradient which is built up by the evaporation of the faster-evaporating solvent.

**Figure 10:**
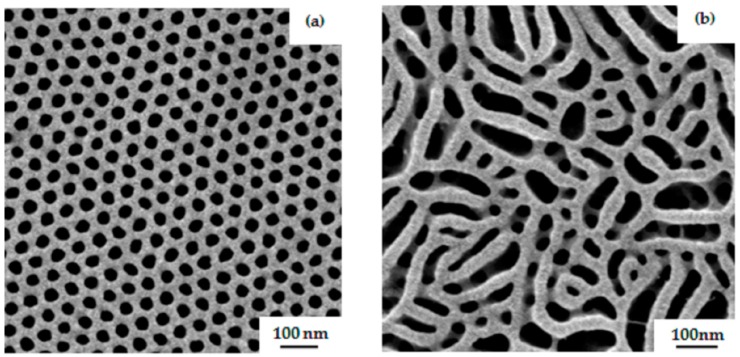
SEM images of surface of the membranes prepared from 22 wt % PS_81_-PGMA_19_^128^ in THF/DMF/DOX (1/1/1); time of evaporation (**a**) 10 s (**b**) 20 s.

It has been reported in the literature that the addition of dioxane (DOX) in the mixture of THF-DMF reduces the solvent quality of the polar block [32]. Therefore, ternary solvent mixtures THF/DMF/DOX for ratios 1/1/1 and 2/1/1, respectively, were studied for their effect on the membrane formation of the PS_81_-PGMA_19_^128^ diblock copolymers. In Figure 10, SEM images are shown of a membrane obtained from a block copolymer solution in THF/DMF/DOX (1/1/1) cast by using a blade height of 200 µm. The viscosity of the ternary solvent polymer solution was approximately comparable to the binary solvent polymer solution, as a lower concentration of diblock copolymer was used. At an evaporation time of 10 s, regular patterns of hexagonally-oriented cylindrical pores were observed which changes to thermodynamically more favorable laying cylindrical morphology by increasing evaporation time for the same reasons as discussed before.

### 3.3. Dynamic Contact Angle Measurement

**Figure 11:**
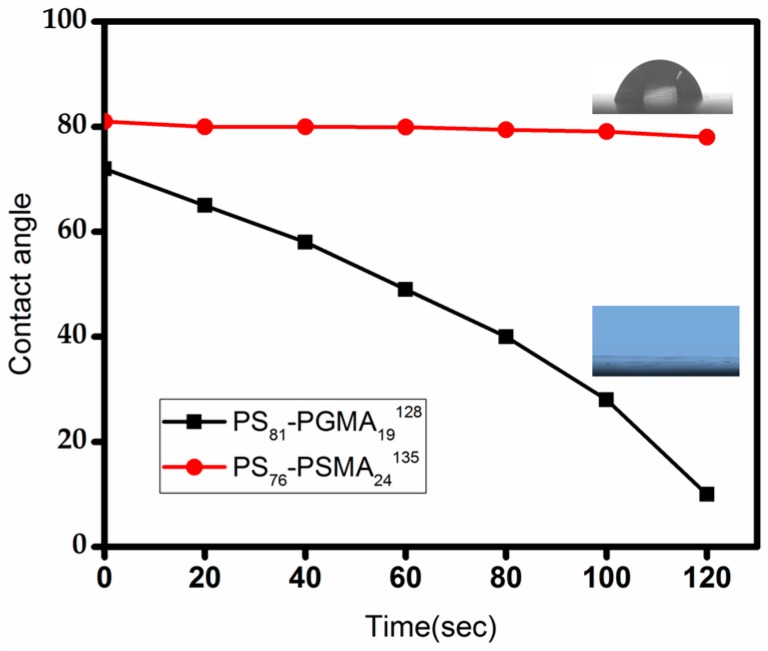
Graphical representation of dynamic contact angle measurement of water droplets (5 µL) onto membrane surfaces developed from block copolymers PS_76_-PSMA_24_^135^ and PS_81_-PGMA_19_^128^ (previously shown in Figure 9a,b).

Hydrophilic and hydrophobic surface properties of block copolymer membranes were characterized by contact angle measurements. Figure 11 shows the variation of the dynamic contact angle with time of 5 µL water droplets on the surface of PS_81_-PGMA_19_^128^ and PS_76_-PSMA_24_^135^ membranes. In case of the PS_81_-PGMA_19_^128^ membrane, the water droplet penetrates into the membrane within 120 s, whereas PS_76_-PSMA_24_^135^ membranes showed no sinking of a water droplet at all, even after 120 s. These results proved that the inner surface of pores is covered by PGMA blocks in the case of PS_81_-PGMA_19_^128^ membranes, which are more hydrophilic than PSMA.

### 3.4. Water Flux Measurement

**Figure 12:**
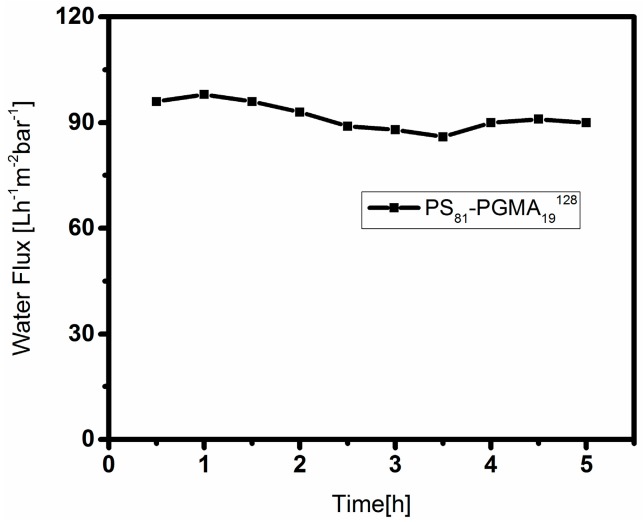
Time-dependent water flux measurements of membrane (previously shown in Figure 9b).

In order to check the applicability of this membrane, time-dependent water flux measurements were carried out in dead-end mode at room temperature shown in Figure 12. The membrane showed low but constant water flux, even after 5 h. This indicates a rather fast swelling of the pore-forming hydrophilic blocks in water, which then hinder the water flux. An additional reason for the low water flux may be the rather dense substructure of the membrane. Membranes of PS-*b*-PSMA showed almost no flux due to completely closed morphology.

## 4. Conclusions

In this paper, we presented a comparative study of amphiphilic PS-*b*-PGMA and hydrophobic PS-*b*-PSMA diblock copolymers for the development of isoporous integral asymmetric membranes. PS-*b*-PSMA diblock copolymers of different composition were successfully synthesized by sequential living anionic polymerization followed by hydrolysis, leading to PS-*b*-PGMA diblock copolymers with overall molar masses in the range of 128–200 kg/mol and low polydispersities *D* = 1.02−1.06. By utilizing the binary THF/DMF and ternary THF/DMF/DOX solvent system, integral asymmetric membranes of PS-*b*-PGMA via self-assembly and non-solvent-induced phase separation process could be achieved as determined by scanning electron microscopy. Within this study, no suitable solvents were found to prepare also isoporous membranes from the PS-*b*-PSMA block copolymer. This will require a more subtle choice of solvents, as the level of selectivity of a solvent and also non-solvent is much less pronounced in a block copolymer composed of similar polar (or nonpolar) blocks. The successfully prepared amphiphilic block copolymer membranes open new horizons for the post-functionalization of membranes due to the presence of two neighbored hydroxyl groups per unit in the pore-forming polymer blocks.

## Figures and Tables

**Table 1:** Characterization data of PS-*b*-PSMA and PS-*b*-PGMA diblock copolymers.

Before Hydrolysis			After Hydrolysis		
Polymer	*M*_n_ (kg/mol)	PDI	Polymer	*M*_n_ (kg/mol)	PDI
PS_76_-PSMA_24_^135^	135	1.03	PS_81_-PGMA_19_^128^	128	1.06
PS_81_-PSMA_19_^170^	170	1.04	PS_85_-PGMA_15_^158^	158	1.07
PS_76_-PSMA_24_^200^	200	1.03	PS_81_-PGMA_19_^190^	190	1.05

**Table 2:** Solubility parameters of solvents and homopolymers. DMF: *N*,*N*-dimethylformamide.

Polymer	δ (MPa^0.5^)	Solvents	δ (MPa^0.5^)
PS	18.6	DMF	24.8
PSMA	19.98	THF	20.3
PGMA	25.8	Dioxane	20.5

## Supplementary Materials

The following are available online at www.mdpi.com/2073-4360/9/6/216/s1.
