# Supplementary material for: Block Copolymer Membranes from Polystyrene-b-poly(solketal methacrylate) (PS-b-PSMA) and Amphiphilic Polystyrene-b-poly(glyceryl methacrylate) (PS-b-PGMA)
Source: Polymers (Basel). 2017 Jun 10;9(6):216. doi: 10.3390/polym9060216 (PMC6432498; doi:10.3390/polym9060216)
Supplement: Supplementary file 1 [file polymers-09-00216-s001.pdf]

Supplementary Materials: Block Copolymer Membranes from Polystyrene-*b*-poly(solketal methacrylate) (PS-*b*-PSMA) and Amphiphilic Polystyrene-*b*-poly(glyceryl methacrylate) (PS-*b*-PGMA)

Sarah Saleem, Sofia Rangou, Clarissa Abetz, Brigitte Lademann, Volkan Filiz and Volker Abetz

Due to difference in solubility behaviour of polystyrene-*b*-poly(solketal methacrylate) (PS-*b*-PSMA) and polystyrene-*b*-poly(glyceryl methacrylate) (PS-*b*-PGMA), the hydroxyl groups of the polymers PS-*b*-PGMA were protected with *tert*-butyldimethylsilyl chloride in the presence of DMF and imidazole at room temperature. Following is the detailed characterization with GPC and ^{1}H-NMR.

Reaction Scheme:

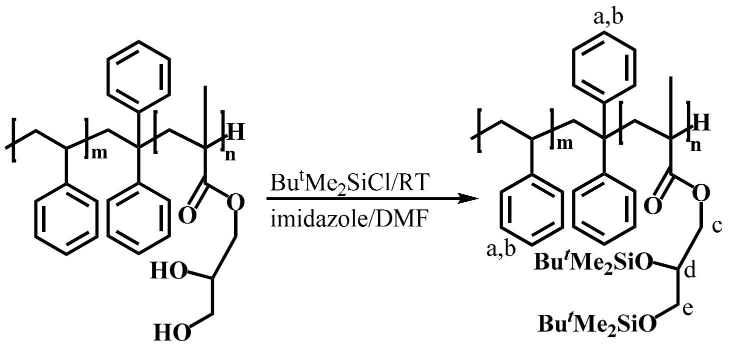

Figure S1. Route of synthesis of PS-*b*-(Bu^{t}Me$_2$Si-PGMA).

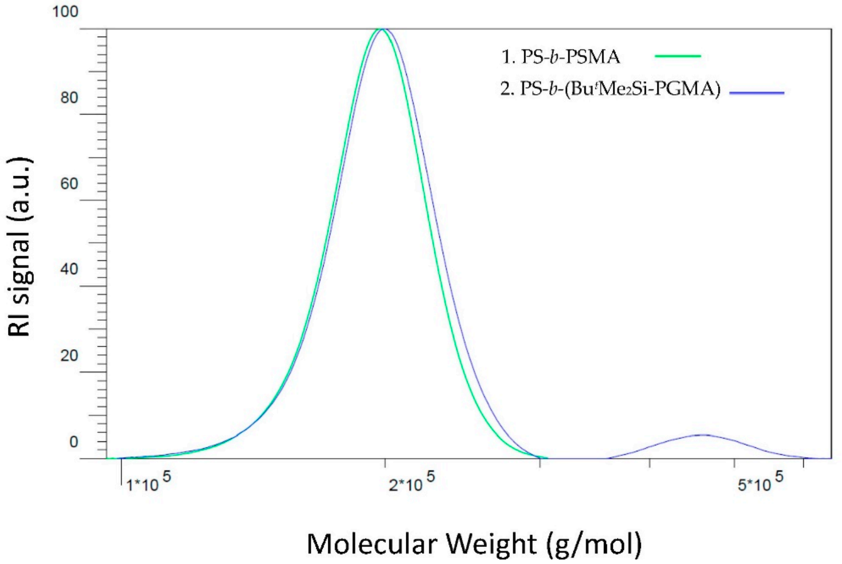

Figure S2. GPC traces (measurement in THF at 30°C using PS standards): (1) PS-*b*-PSMA; (2) PS-*b*-(Bu^{t}Me$_2$Si-PGMA).

Table S1. Characterization of PS-*b*-PSMA and PS- *b*-(Bu^tMe_2Si-PGMA) diblock copolymers.

Polymers *	Mn (kg/mol) **	Mn (kg/mol) ***	PDI
PS_{83}-*b*-$PSMA_{17}$	176	191	1.02
**** PS_{87}-*b*-$PGMA_{13}$	167	-	-
PS_{84}-*b*-$(Bu^tMe_2Si\text{-}PGMA)_{16}$	174	199	1.05

* lower case numbers indicate weight fraction (%) of the blocks; **molecular weights from ^{1}H-NMR based on PS-precursor molecular weight from GPC; ***apparent GPC molecular weights; **** Insoluble polymer in THF, for this reason molecular weight is calculated by PS precursor (measurement in THF at 30°C) in combination with weight percentages derived from ^{1}H-NMR in DMF *d7*, assuming 100% conversion.

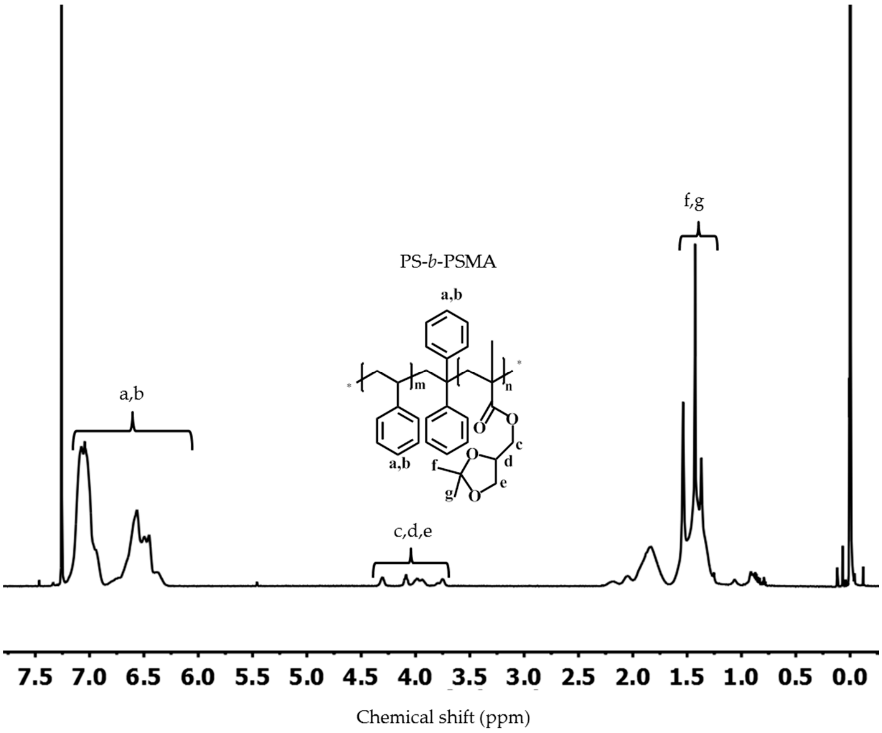

Figure 3. ^{1}H-NMR spectra of PS-*b*-PSMA in $CDCl_3$.

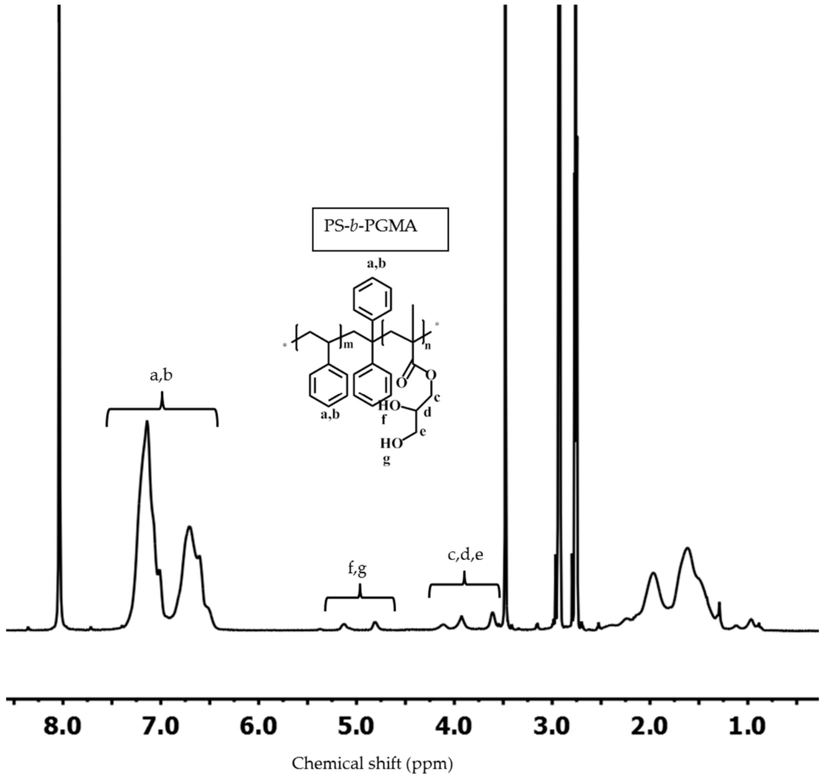

Figure S4. ^{1}H-NMR spectra of PS-*b*-PGMA in DMF-*d7*.

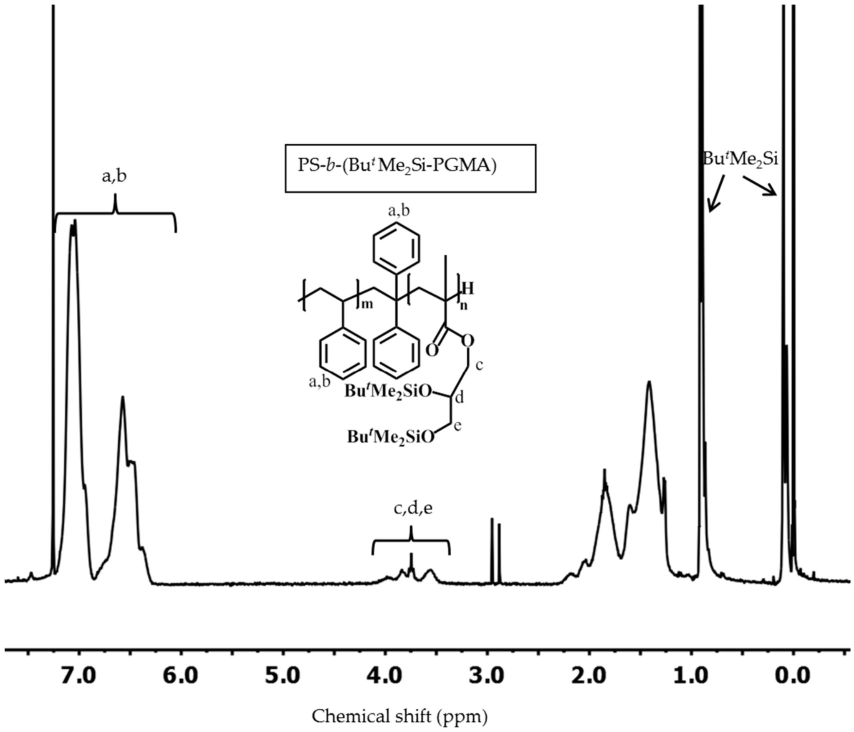

Figure S5. ^{1}H-NMR spectra of PS-*b*-(Bu^{t}Me$_2$Si-PGMA) in $CDCl_3$.
